# The Dentist

**Published:** 1897-12

**Authors:** B. F. Sheppard


ARTICLE VIII.

### THE DENTIST.

BY DR. B. F. SHEPPARD.

The dentist should be a man of decision and self-reliance. He should feel that he knows, and is able to do, so that his very attitude will forbid the suggestions of those who will have everything their own way, and still hold him responsible for the success of the operation. Many a man who really knows fails because of a lack of self-reliance, thoroughness and stamina equal to his knowledge. He should cultivate that spirit of fortitude which will enable him to grapple with all difficulties bravely, decisively, marking the hand of a master in all his work.

The dentist should have combativeness and destructiveness sufficient to give efficiency, enabling him to witness pain and suffering and to emp oy the means necessary to relieve without trembling or hesistancy. It has been said that the physician needs the lion's heart and the woman's hand. It is equally true of the dentist. He should have combativeness and destructiveness, firmness and self-esteem, to give the lion-like power, ideality, constructiveness and gentleness, which come from refinement of temperament, to give the woman's hand. We have seen men who had power, self reliance and persistency, who carried these forces with admirable gentleness, and self-control.

He should have prudence, caution, secretiveness and good common sense, and the more common sense the better. Cautiousness will make him desire to do the right thing at the right time and place. Secretiveness will enable him to keep his mouth shut at the proper time, and control his countenance as well as his expressions, and be always master of the situation, when too open an expression would have been disastrous.

He should have large hope and mirthfulness, and aa excellent talking talent, so that he may put an atmosphere of mirthfulness and good feeling around im at all times.

Large constructiveness is necessary to the dentist, in fact his profession has been considered by some as only a mechanical trade. While this is not true, yet his ability to put together, his judgment or knowleage of the right condition in constructions, will mark his success in prosthetic and operative dentistry, as well as all operations in oral surgery. It will enable him to meet the requirements of anæsthetics in dentistry, elaborating and bringing together the many parts into one harmonious whole.

He should have strong social feeling, so that all the relations of social life may be appreciated by him; so that his patients will become friends, and feel a lively interest in his success.

He should have continuity of purpose. All the great minds of the world have been marked by a spirit of continuance, a pushing ahead, and an unflagging zeal which could brook nothing less than ultimate success.—*Ohio Journal.*

